# A juvenile case of conjunctival atypical nevus

**DOI:** 10.1186/1746-1596-8-64

**Published:** 2013-04-22

**Authors:** Cristina Colarossi, Mario Milazzo, Milena Paglierani, Daniela Massi, Lorenzo Memeo, Vincenzo Canzonieri

**Affiliations:** 1 Pathology Unit, Department of Experimental Oncology, Mediterranean Institute of Oncology, Via Penninazzo 7, Viagrande, CT, Italy; 2 Gecas srl, Via San Paolo 68, Gravina di Catania, CT, Italy; 3 Division of Pathological Anatomy, Department of Critical Care Medicine and Surgery, University of Florence, Viale GB Morgagni 85, Florence, Italy; 4 Division of Pathology, CRO - Centro di Riferimento Oncologico, Istituto Nazionale Tumori, Via Franco Gallini 2, Aviano, PN, Italy

## Abstract

**Virtual slides:**

The virtual slide(s) for this article can be found here: http://www.diagnosticpathology.diagnomx.eu/vs/2973228795724608

Melanocytic nevi are the most common tumors of the conjunctiva, accounting for 28% of all neoplastic lesions. These tumors, despite their benign behavior, share some atypical histological features with nevi found in other anatomic sites like the genital and acral regions, globally designated as nevi with site-related atypia. Moreover, in children and adolescents, rapidly growing conjunctival nevi show sometimes worrisome histological patterns in association with a prominent inflammatory infiltrate that may lead to diagnostic problems. In this paper we describe a juvenile compound nevus characterized by marked melanocytic atypia and severe inflammation, which can be considered a rare case of juvenile conjunctival atypical nevus. The final diagnosis relied on morphological and immunohistochemical characterization of the large epithelioid melanocytic cells, and on the results of FISH analysis.

## Background

Among conjunctival tumors, melanocytic lesions represent 53% of all excised conjunctival lesions [1]. Nevi are the most common, found in 52% of the cases, followed by melanoma in 25%, primary acquired melanosis (PAM) in 21%, and racial melanosis in 3% [2]. Similarly to when they occur in the skin, nevi can be congenital, if they appear at birth or within the first 6 months of life, or acquired, if they become clinically evident in the first or second decade of life as discrete, variably pigmented, slightly elevated lesions, frequently containing clear cysts. Nevi derive from a benign proliferation of melanocytes in the basal layer of the conjunctival epithelium and are classified, like on the skin, as junctional, compound, and subepithelial, the compound being the most common pattern. Subepithelial components are frequently hyper-cellular and may have cytologic atypia, but are usually associated with symmetry, demarcation from the surrounding stroma and melanocytic maturation, all hallmarks of a benign lesion with reassuring findings [3]. Nevi are typically located in the interpalpebral bulbar conjunctiva (67-72%), especially the interpalpebral area, followed by the caruncle (15%-22%) and tarsus (0.7%). The vast majority of nevi in these sites are benign with the exception of tarsal melanocytic lesions that are frequently regarded as suspicious for malignancy. On the other hand, conjunctival melanomas typically arise in adults (median age 62 years), but rare cases of conjunctival melanoma in children have been recognized [4].

Here we report a case of juvenile conjunctival nevus, whose histology was uncommonly atypical, not only in the subepithelial area, but also deeply within the lesion, thus raising the suspicion of malignancy. This was an entity that has, so far, drawn little attention in the literature. Consequently, accurate morphological and immunohistochemical studies were performed to render a final diagnosis of atypical nevus, and pertinent FISH analysis gave interesting results to support that it was of a benign nature.

## Case presentation

**Figure 1:**
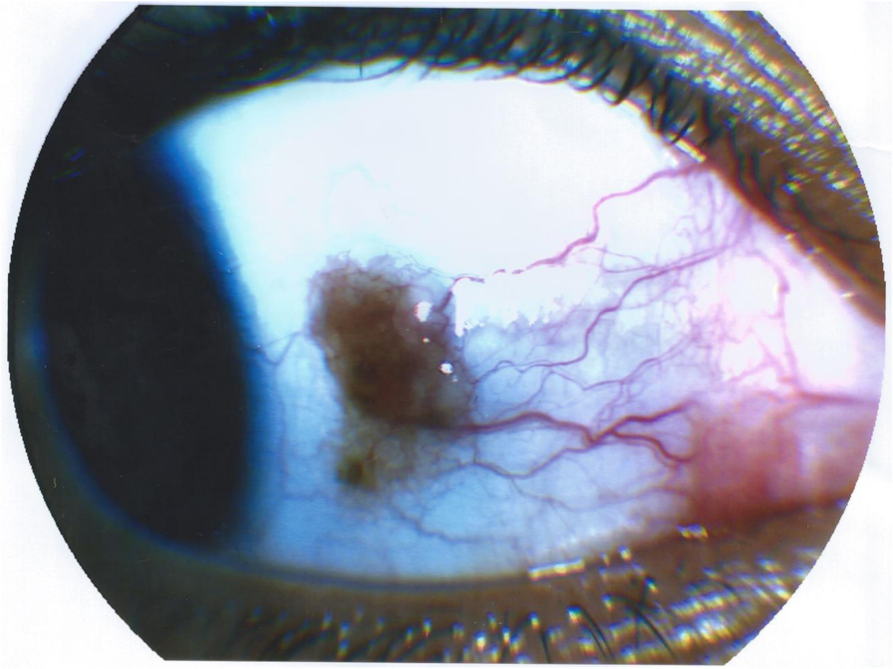
Gross photography of the pigmentated lesion with slight irregular borders.

**Figure 2:**
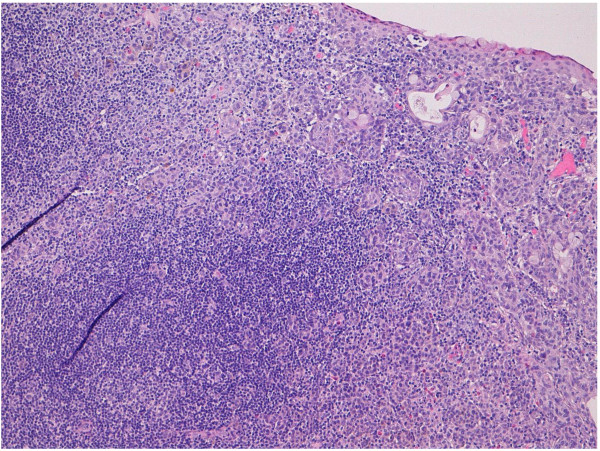
Deep compound lesion with dense inflammatory infiltrate and small glandular cysts (H&E 10x).

**Figure 3:**
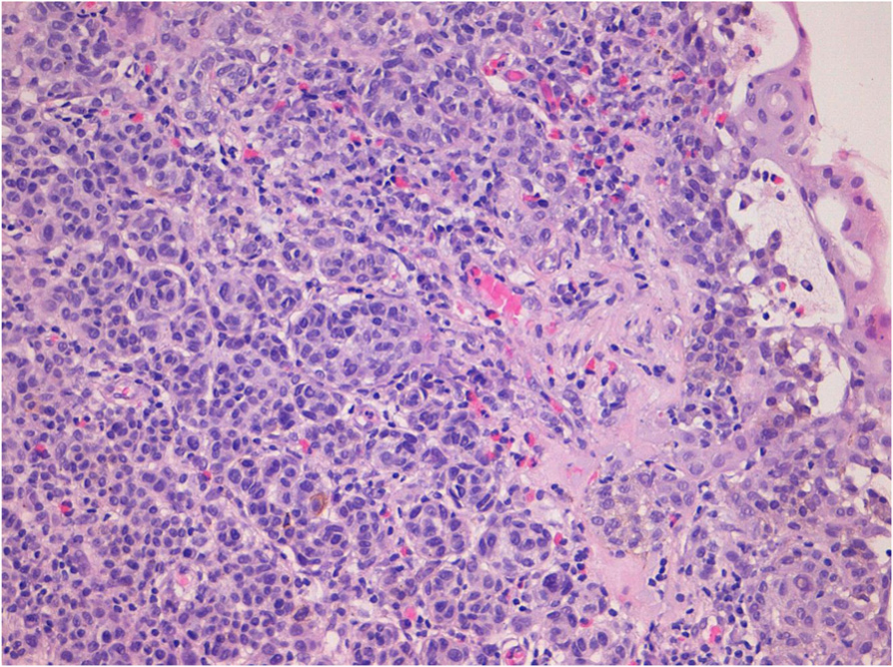
Atypical nevus cells with small glandular cysts (H&E 20x).

**Figure 4:**
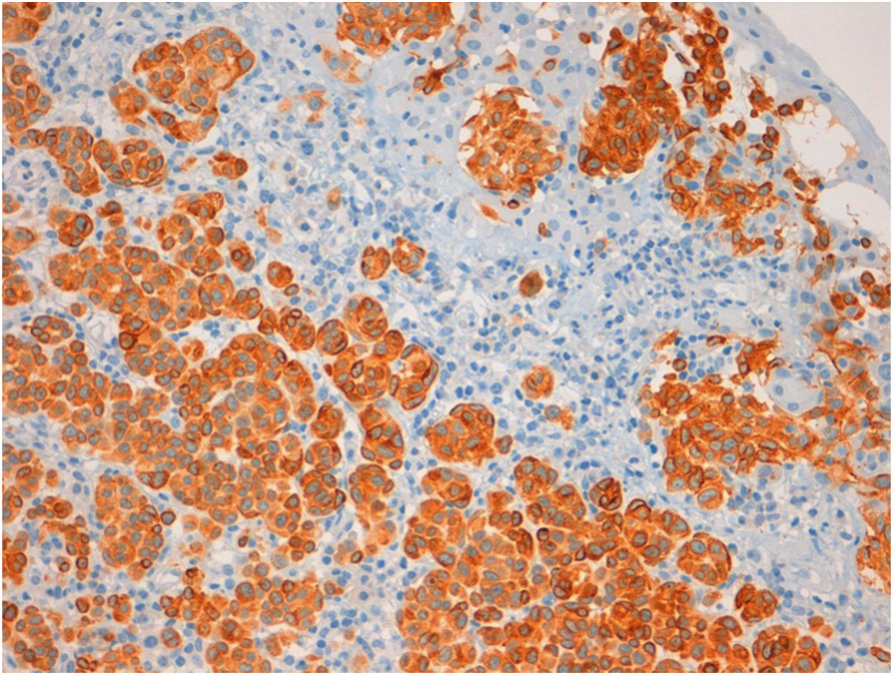
Melan-A expression in atypical nevus cells (20x).

**Figure 5:**
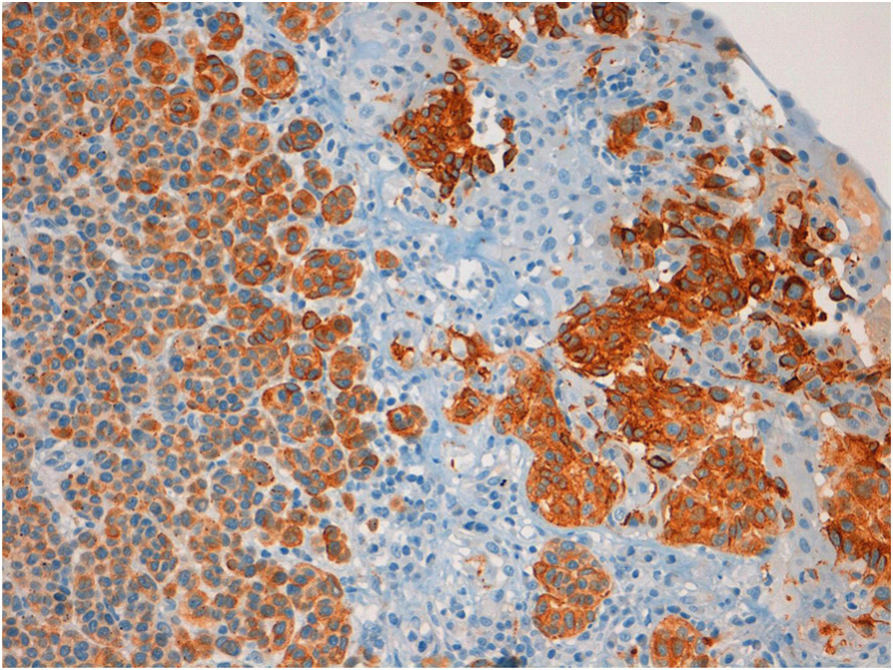
HMB-45 expression in atypical nevus cells (20x).

**Figure 6:**
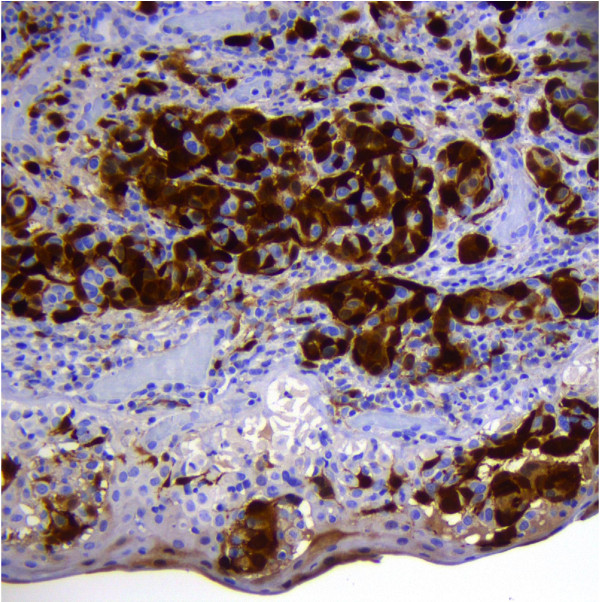
p16 expression in atypical nevus cells (20x).

A thirteen-year-old female patient was admitted at our institution with a bulbar juxtalimbal conjunctival pigmented lesion (Figure 1). She underwent an excisional biopsy and the specimen was formalin-fixed and paraffin-embedded for routine examination. The lesion was 3 mm at its widest point with well demarcated borders. A prominent and dense infiltrate of inflammatory cells was present throughout the lesion, including formation of follicles. Small cystic dilatation of the conjunctival gland was only focally observed (Figures 2 and 3). High power examination showed a compound melanocitic proliferation with altered maturation sequence and deep nests of atypical, large, and faintly pigmented epithelioid nevus cells with nuclear pseudo-inclusions in the substantia propria. No evidence of mitotic activity was observed. The junctional component showed focal pagetoid spread of melanocitic cells in the epithelium, as clearly visualized with Melan-A (Figure 4) and HMB-45 (Figure 5) antibodies. Ki-67 positivity was mainly present in nevus cells within the epithelial-stromal junctional zone and an overall mean proliferation index of 2% was calculated. Immunohistochemical analysis showed a diffuse nuclear and cytoplasmic expression of p16 protein (Figure 6).

**Figure 7:**
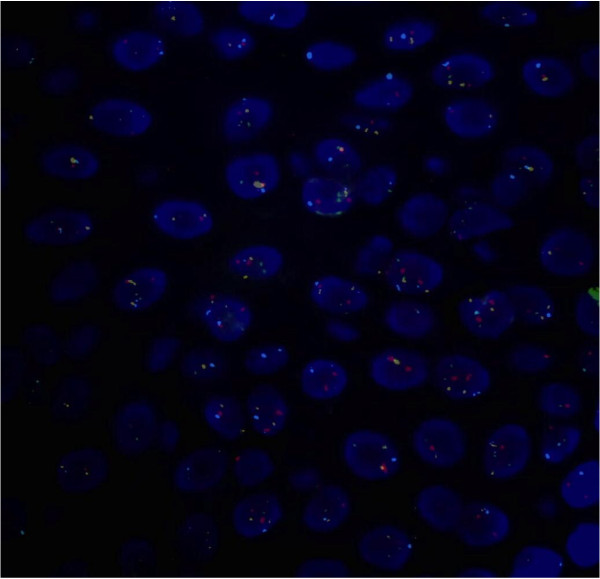
Fluorescence in situ hybridization, (FISH) shows a normal chromosomal pattern with two signals of each probe: CCND1(11q13) SpectrumGreen, RREB1 (6p25) SpectrumRed, MYB (6q23) SpectrumGold  and  centromere of chromosome 6 SpectrumAqua (100x).

FISH probes were used on four loci: RREB1 at 6q23, 6 centromere, MYB at 6p25 and CCND1 at 11q13, (Vysis Melanoma FISH Probe Kit, Downers Grove, Illinois, USA). FISH analysis of the lesion did not show genetic aberrations, which allowed for a diagnosis of atypical compound nevus (Figure 7).

## Discussion

In this report we present a challenging case of conjunctival compound nevus in a young female patient. The atypical histological features that characterize the lesion were what made this case peculiar. The lesion could be included in the spectrum of atypical lesions associated with the concept of “site specific atypia”, but this approach is not fully convincing, in part due to the severe inflammation - like a host reaction to malignancies - that was present in our case. Moreover, we observed nests of atypical large cells at the base of the nevus that showed no maturation activity, hence, the differential diagnosis with melanoma became the main diagnostic problem. The features that could have ruled out the diagnosis of melanoma were the sharp circumscription of the lesions and the lack of mitotic activity in the substantia propria.

In general, the patient’s young age and the cystic nature of the lesions are consistent with benign behavior, so that the vast majority of these cases are diagnosed as inflamed juvenile conjunctival nevi [5], taking also into account that conjunctival malignant melanoma is extremely rare in the first two decades of life.

As far as our case is concerned, the rarity of cystically dilated glands, the marked atypia of the lesional cells and the focal pagetoid spread, suggesting, per se, worrisome histological findings, were in keeping with the diagnosis of atypical nevus. Ki-67 findings were not significantly increased if compared to normal conjunctiva [6] Immunohistochemical results concerning p16 were in line with our histological findings. p16 protein regulates the cell cycle and plays a major role in melanomagenesis, and is expressed in several types of benign melanocytic nevi [7]. Moreover, p16 immunohistochemical expression is helpful in some cases to differentiate between childhood nodular Spitzoid melanomas and Spitz nevi [8]. In addition, FISH analysis was performed for diagnostic purposes. In fact, in recent papers, the application of fluorescence in situ hybridisation has been demonstrated to be a useful tool for the diagnosis of ambiguous melanocytic neoplasms of the conjunctiva [9,10] as demonstrated in skin lesions [11,12]. Taking into account that the sensitivity of the technique is around 87%, a positive FISH result provides additional support in favor of melanoma, whereas a negative FISH result makes melanoma less likely. In our case, FISH analysis, targeting 6p25, 6q23, 11q13 and centromere 6, did not show any genetic aberration and supported our diagnosis of atypical nevus.

The interest of this particular case relies on the differential diagnosis between atypical benign nevic lesions and melanomas in the conjunctiva, using a multi-level evaluation of morphologic, immunohistochemical and in situ molecular findings.

## Consent

Written informed consent was obtained from the patient for publication of this case report and any accompanying images. A copy of the written consent is available for review by the Editor-in-Chief of this journal.

## Competing interests

The authors declare that they have no conflict of interest.

## Authors’ contributions

CC conceived of the study, participated in its design and drafted the manuscript. MM participated in the design of the study. MP carried out the FISH analysis. DM participated in the design of the study and drafted the manuscript. LM participated in the design of the study and drafted the manuscript. VC participated in the design of the study and drafted the manuscript. All authors read and approved the final manuscript.
